# Co-designing a Mobile Gamified Attention Bias Modification Intervention for Substance Use Disorders: Participatory Research Study

**DOI:** 10.2196/15871

**Published:** 2019-10-03

**Authors:** Melvyn Zhang, Sandor Heng, Guo Song, Daniel SS Fung, Helen E Smith

**Affiliations:** 1 National Addictions Management Service Institute of Mental Health Singapore Singapore; 2 Department of Developmental Psychiatry Institute of Mental Health Singapore Singapore; 3 Family Medicine and Primary Care Lee Kong Chian School of Medicine Nanyang Technological University Singapore Singapore

**Keywords:** attention bias, cognitive bias, gamification, participatory design research, psychiatry, apps, cognitive bias, mobile intervention

## Abstract

**Background:**

Advances in experimental psychology have highlighted the need to modify underlying automatic cognitive biases, such as attentional biases. The effectiveness of bias modification has been well studied for substance use disorders. With recent advances in technology, it is now possible to work outside the laboratory with Web-based and mobile-based attention bias interventions. Gamification technologies might also help diminish the repetitiveness of the task and increase the intrinsic motivation to train. The inconsistent findings of the impact of gaming on the effectiveness of mobile interventions call for further work to better understand the needs of patients (users) and health care professionals.

**Objective:**

The aim of this study was to involve patients, together with health care professionals, in the design of a gamified mobile attention bias modification intervention for substance use disorders.

**Methods:**

The participatory design research method adopted is that of a user-oriented design approach in the form of a future workshop. In the first phase of the workshop, participants shared their critique of an attention bias modification intervention. In the second phase of the workshop, participants were asked to brainstorm features. Participants were also shown gamification approaches and asked to consider if gaming elements could enhance the existing app. In the last phase, participants were asked to sketch a new prototype.

**Results:**

Three co-design workshops were conducted with health care professionals, inpatients, and outpatients. There were 20 participants, consisting of 10 health care professionals and 10 patients. When asked to identify the limitations in the existing app, common issues identified were those of the design, visual probe task, and the included images. Outpatients were also concerned with the safety of administration of the intervention. In the brainstorming sessions, health care professionals made recommendations as to how the stimulus, the mechanism of responding, and the presentation of the scores could be enhanced. Inpatient participants recommended the addition of functionalities, such as information on the harms associated with the substance use, and for there to be enhancements in the design, images, and task. Outpatient participants perceived a need to improve the images and presentation of the results and recommended the inclusion of gaming features. There were differences in opinion on the inclusion of gaming features, as only health care professionals endorsed their inclusion. In the last phase of the workshop, participants were tasked with the conceptualization of prototypes, and the commonality in the design was for a gradual shortening of the interval for stimulus/image presentation.

**Conclusions:**

The results from this research will guide the development of an app that meets the specific needs of patients and is still based on a pre-existing validated task paradigm.

## Introduction

Illicit substances like opioids, cannabis, and stimulants are highly abused worldwide [1]. Unfortunately, there are limited pharmacological approaches to help the affected individuals achieve and maintain abstinence, which is crucial because of the potentially severe complications such as medical comorbidities and death [2,3]. Therefore, the mainstay of management is psychological therapies. Although cognitive behavioral therapy is used often, its effectiveness is variable and not always sustained; some studies reported that 40%-50% of individuals relapsed in the first year and 70% relapsed within 3 years [4]. It appears that conventional psychotherapies do not address all the etiological factors; hence, individuals relapse back to addiction. Moreover, a recent bibliometric review has highlighted a decline in the growth trend for psychotherapies applied for substance use disorders [5]. Advances in experimental psychology have informed the dual-process theoretical model [6,7], which postulates that existing therapies only address the cognitive control processes, but not the underlying automatic, unconscious processes. The dual-process theoretical model suggests there are two common automatic processes occurring in individuals with addictive disorders: attention bias and approach bias. Attention bias refers to the preferential allocation of attentional processes toward substance-related cues [8], whereas approach bias refers to the automated tendencies for individuals to seek out and reach for substance-related stimuli [9]. To assess these unconscious biases, tasks like the Stroop task and the Visual Probe task are commonly used [10]. These tasks are also used for bias modification, which focuses on retraining the biases, so that attention is directed away from the stimulus of interest [10].

The effectiveness of bias modification has been well studied. Cristea et al [11] reported moderate effectiveness (Hedges G=0.60) of cognitive bias modification in people with alcohol and tobacco use disorders. Other studies in clinical settings have similarly reported that bias modification helps reduce biases and other positive outcomes [9,12]. Most interventions included in these reviews were delivered in a laboratory setting [13], but with recent advances in technology, it is now possible to work outside the laboratory with Web-based and mobile-based interventions and provide early interventions and psychoeducation for addictive disorders [14]. Our recent review synthesized evidence for mobile attention bias interventions [15] and highlighted seven studies demonstrating effectiveness. Use of gamification technologies in these mobile interventions is the next advancement, as gamification might help diminish the repetitiveness of the task and increase both the extrinsic and intrinsic motivation to train [16]. Interestingly, in a review on gamified attention bias apps, only half of the studies adopting a gaming approach were more effective than standard care [17].

These inconsistent findings of the impact of gaming on the effectiveness of mobile interventions calls for further work to better understand the needs of patients (users) and health care professionals (providers). Participatory action research methods are a “systematic inquiry, with the participation of those affected by the problem being studied, for education and action or effecting social change” [18] and are well suited to the development of relevant interventions for the end user. Workshops and focus groups are the most common methods of cocreation, and these techniques are increasingly used in medicine. In our review of how participatory action methods have been applied for technological interventions in psychiatry [18], we reported seven studies describing how these methods have been used in the fields of perinatal depression, dementia, self-harm, and general mental health or youth mental health issues [18]. Such methods help in exploring perceptions and refining the existing task; they could also help researchers understand the reasons underlying the diminishing motivation and interest in such interventions [18]. The prior review focusing on participatory action research [18] highlighted the need to apply these methods for bias modification intervention research. Moreover, Zhang et al [19] also reported at least 17 bias modification apps in the commercial store, but only one app had an academic input. Similarly, in their review of smoking cessation apps, Haskin et al [20] found that only two of the validated apps were amongst the top 50 apps in the app store. It is evident from the review that there is a great divide between academics, developers, and the end users (or patients). Including methods of participatory design research will help bridge this disconnect between different stakeholders.

This study aimed to involve patients, together with health care professionals, in the design of a gamified mobile attention bias modification intervention. We wished to address four questions from the perspectives of health professionals and patients: (1) What are participant’s perspectives of the mobile attention bias modification intervention? (2) What features of a mobile attention bias modification intervention would a participant expect, to help minimize attrition from the task and increase both intrinsic and extrinsic motivation in completing the intervention? (3) Would gamification (ie, application of gaming elements) help in enhancing the existing mobile bias intervention task (ie, increase the magnitude of bias change, increase motivation, and reduce rates of attrition)? (4) What gaming elements are preferred?

## Methods

### Design Approach

The approach adopted was that of a Future Workshop, a method deemed to be optimal for the generation of new, innovative ideas [21]. We planned to conduct three co-design workshops: one with health care professionals, one with inpatients, and one with participants from outpatient settings.

### Study Settings and Recruitment

Participants were recruited from the largest addiction treatment center in Singapore that specializes in both substance and behavioral addictions. The health care professionals recruited were addiction psychiatrists and addiction counsellors. The number of health care professionals and patients recruited was 10 for each group, so that there was equal representation from both groups of participants. In addition, patients who were at different stages of recovery were invited to participate, to ensure that the collated perspectives would not be biased. Informed consent was obtained in accordance with the Human Biomedical Research Guidelines from all participants.

### Participants

Patients were included in the study if they were aged between 21 and 65 years; diagnosed with a primary psychiatric disorder of alcohol, opioid, cannabis, stimulant use disorder; able to speak and write in English; and knew how to use a smartphone device. Patients were excluded from the study if they had a significant psychiatric comorbidity (moderate to severe depressive disorder, anxiety disorder, psychotic disorder), were non–English-speaking, or had an existing cognitive impairment or intellectual disability. The inclusion criteria for selecting the patients were similar to those of our prior feasibility study [22] and representative of the demographics and clinical characteristics of patients admitted for treatment in our program.

For health care professionals to be included in the study, they had to be currently working in an addiction unit and actively involved in the treatment of individuals with addictive disorders with a minimum of 2 years of experience working with clients with addictive disorders.

### Workshop Procedures

All participants completed a questionnaire prior to participating in the co-design workshop comprising of three phases. The questionnaires included questions on the demographics of the health care professionals and demographics and clinical characteristics of the patients. The principal investigator (MZ), a psychiatrist specializing in addiction medicine, facilitated all three workshops.

#### Phase 1

**Figure 1:**
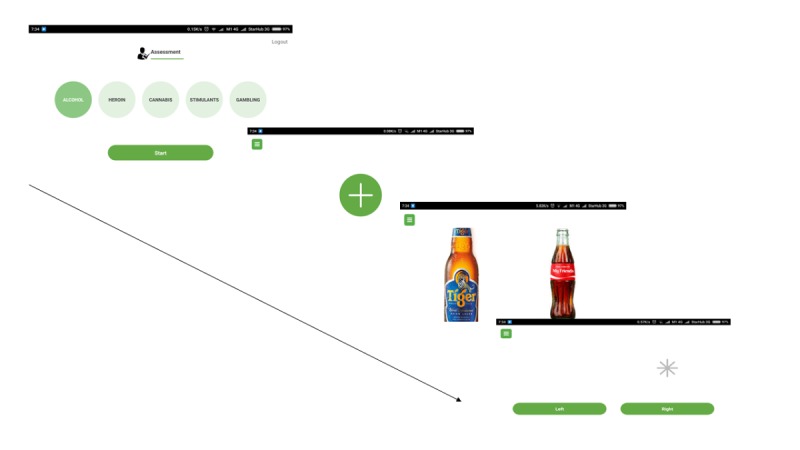
Overview of the existing mobile attention bias intervention.

In the first phase of the co-design workshop, individuals critiqued the existing smartphone-based attention bias modification intervention. The existing attention bias modification intervention was based on a prior protocol [23]. In the mobile app, participants were able to select the intervention specific for their substance of abuse. In the assessment task, participants were presented with a central fixation cross. Following the disappearance of the cross, both a neutral stimulus and a nonneutral stimulus were presented simultaneously. A probe would then replace either of the stimuli, and individuals were to indicate the position of the probe, within a predetermined response time. In the assessment task, the probe would replace either stimulus equally. In the intervention task, the probe would replace the nondrug stimulus all the time. Figure 1 provides an overview of the nature of the assessment and intervention tasks.

Participants were introduced to the rationale of the research project and the objectives of the study. Participants were then shown a presentation of the existing attention bias modification app. They were able to try the app on the provided tablet devices. Participants were asked to provide their thoughts narratively and identify limitations. The questions posed were as follows: (1) Having seen and used the existing app, what are your thoughts about it? (2) What are some of the limitations of the current app?

#### Phase 2

In the second phase, individuals brainstormed for solutions to their critiques. Participants were asked to brainstorm features that could be added to the existing mobile app. These questions were as follows: (1) What additional features do you think could be added to the app? (2) Why do you think that these additional features will be helpful? Participants were encouraged to write down their ideas on sticky notes, following which these notes were collated by members of the study team and discussed amongst the whole group.

As one of the objectives was to determine whether gamification could address some of the limitations of the existing app, participants were shown screenshot examples of some of the attention bias commercial apps, which included elements of gamification [19]. Gamification elements, ie, digital rewards, avatar and competition, feedback, leader board, time pressure, and 3D environment, were shown to participants. To ensure everyone had some understanding of gamification techniques, the facilitator provided an overview of techniques summarized in the literature (Table 1) [24]. Participants were shown gaming strategies adopted in commercial apps and approaches used for apps in other disciplines and asked to consider strategies that are appropriate for the current intervention.

**Table 1:** Overview of gamification approaches [24].

Gaming approach		Description
**Economic gamification techniques**		
	Marketplace and economies	Providing gamers with a virtual currency that allows them to deal in game
	Digital rewards	These include badges, game currency, game points, virtual goods, and powers or abilities
	Real-world prizes	Provides gamers with options to exchange in-game credits for real-world prizes such as vouchers or other forms of goods and services
**Social gamification techniques**		
	Avatar	Allows individuals to choose a virtual character to represent oneself
	Agent	A virtual character that help guides or provides instructions to user
	Competition	Allows individuals to compete with other players or with each other
	Teams	Game that involves several individual players, allowing them to interact and form relationships
	Parallel communication systems	Allows individuals to communicate with one another
	Social pressure	Ability of game to pressurize individuals to perform in certain task, so that he or she will be invited to subsequent events.
**Performance-orientated techniques**		
	Feedback	Spoken, visual, or auditory feedback about user’s performance
	Levels	Information on the stage of a game one has attained
	Secondary game objectives	Secondary goals that reward the player upon completion
	Ranks of achievement	Measurement of character development
	Leaderboards	Allows for comparisons with other players
	Time pressure	Pre-determined time limits for task completion
**Embedding-focused techniques**		
	Narrative context	A storyboard or stories that guide development of the character
	3D environment	3D models of objects that parallel the real world

Participants were asked to discuss their perspectives about the gamification ideas described and then individually select their top three gamification techniques that they felt were most appropriate to be applied in the existing app.

#### Phase 3

In the last stage of the workshop, participants were divided into smaller groups of two to five to develop frame-by-frame sketches of a prototype app that incorporated the solutions that they have proposed. Participants sketched freely on the paper provided by using a variety of writing instruments provided. They were told to include the original task but could modify it. All the groups were given 15 minutes to work on this task.

Prior to the completion of the workshop, participants were also shown a set of substance images that have been incorporated since into the existing app. Participants were assigned to rate the relevance of the images for an individual with an addictive disorder, on a Likert scale (scores ranging from 1 to 10, where 1 is not relevant and 10 is extremely relevant). Participants were also asked to share their perceptions with the facilitator of the workshop.

### Ethical Approval

This study obtained ethical approval from the National Healthcare Group’s Domain Specific Research Board (ethics approval number 2018/01363).

### Data Analyses

Descriptive statistical analysis was performed using SPSS (version 24; IBM Corp, Armonk, New York) for the quantitative data collated from the questionnaire. The workshops were audio-recorded and transcribed verbatim by the principal investigator and independently by a private transcription service (WayWithWords). The principal investigator (MZ) then listened to the audio recordings of the workshop and developed a coding frame. To ensure reliability of the coding frame that was adopted, two authors (MZ and SH) reviewed the transcripts and discussed the coding frame, thus ensuring that the process of intercoder consensus was adhered to [25]. If there were any disagreements, they were resolved through discussion with another author. The codes identified were classified into categories and then reorganized into themes. NVivo (version 12.0; QSR International, Melbourne, Australia) was used to facilitate the analysis. Two independent members of the study team reviewed the sketches of the prototype and identified the common elements.

## Results

### Demographics of Participants

Three co-design workshops were conducted with health care professionals, inpatients, and outpatients. There were 20 participants, consisting of 10 health care professionals and 10 patients (5 inpatients on a detoxification and rehabilitation program and 5 outpatients who were clinically stable and mostly in abstinence from their substance use). The mean age of the health care professionals was 47.3 years, and the mean time caring for individuals with addictive disorders was 12.7 years. Of the 10 health care professionals, 8 were male. In terms of ethnicity, four were Chinese, one was Malay, and five were Indians. The mean age of inpatients was 44.4 years; one had an alcohol use disorder and four had a substance use disorder. For outpatients, the mean age was 43.2 years, and all participants had used both alcohol and illicit substances, except one inpatient. Multimedia Appendix 1 provides further information on the demographic characteristics of patient participants.

### Phase 1

When responding to the existing app, health care professionals identified issues with its design, the visual probe trials, and the images. Both patient groups perceived issues with the visual probe trials and the images included in the app, and the outpatients also commented on the design and safety issues with the administration of the existing app. Table 2 provides a summary of the verbatim comments of the participants in each of the themes identified.

**Table 2:** Themes related to limitations of the existing app.

Themes	Health care professionals (n=10)	Inpatient participants (n=5)	Outpatient participants (n=5)
Design of the app	“Buttons were too small” making them “easy to miss” [Participant 2] “Press a few times, will add on to the reaction time” [Participant 1] “There is no try out to understand how it works.” [Participants 3 and 5]	No mention	“More instructions”[Participant 2] “When I got things clear, I focus on star” [Participant 5] “No design or anything” [Participant 1] Buttons to be “bigger” [Participant 1] “Joystick interface” to indicate a response [Participant 2]
Visual probe tasks	“Too many repetitions” [Participant 2] The task was “so fast” [Participants 3 and 5]. “Distinguish the frame from one another looking at the asterisk” [Participant 1] “Focusing on the asterisk only.” [Participants 3 and 7] “Pictures were so fast, after a while, I stopped paying attention and just look for the asterisk” [Participant 8]	“Were too fast” [Participants 3, 5 and 7] “My mind cannot catch up, fingers cannot catch up” [Participant 5] “Repetition is the same” [Participant 5] Task is “too tedious” [Participant 3]	The trial was “too long” [Participants 1 and 4] “Too fast” and “quick” [Participants 2, 4, and 5] “I never even have a chance to see” [Participant 1] “Too fast. My mind was to aim for the star. The pictures I was not interested.” [Participant 4] “It is too fast that we don’t really see the picture” [Participant 3]
Images included in the visual probe task	“Image colour does not stick out” [Participant 3] “Non-white background will enhance the focus of the images” [Participant 2]	The “colour is dull” and “the images are repetitive” [Participant 1] “I see the same image for 30 times or more” [Participant 1]	“Keep seeing the same pictures” [Participant 3] “Very boring, put more pictures” [Participant 1] “Maybe more picture, very repetitive. I keep on seeing the same pictures over and over again” [Participant 2] “It comes in pairs. It always weed and chocolate cake. You can figure out” [Participant 2]
Safety of administering the app	No mention	No mention	“Triggering. Like I think just keep seeing pictures of drug of choice” [Participant 2] “Early recovery is triggering. For myself, if I am in detox, might trigger me.” [Participant 5]

### Phase 2

When brainstorming features that could be added to the existing mobile app, the health care professionals suggested improvement to the stimuli, the mechanism of responding, and the presentation of the scores. Inpatient participants recommended additional functionalities and enhancements in the design, images, and task. Outpatients recommended improvements in both the design, included images, and the presentation of the results and perceived gaming features to be a solution. Table 3 provides a summary of the verbatim comments of the participants in accordance with the themes identified.

**Table 3:** Themes related to solutions addressing limitations of the existing app.

Themes	Health care professionals (n=10)	Inpatient participants (n=5)	Outpatient participants (n=5)
Images included in visual probe task	“As much as possible, it should be similar as possible. The two pictures should be of the same quality and same size” [Participant 2] “How come the tiger bottle is so big and the cola bottle so small? Obviously, my eyes will zoom to the big one. I am attracted to the obvious. It just shines out” [Participant 5]	“Something associated with alcoholism. Clubbing, coffee shop, anything associated with alcoholism” [Participant 1] Images with substances in different “environment” [Participant 3] “Can include like family members. Drinking with family members” [Participant 2] “Images personalized, can identify with” [Participant 3]	“Higher intensity” of images“ instead of just 2 pictures [Participant 1]
Design of app	“Is it possible to press on the picture instead of right/left. Pictures are bigger than the right/left button” [Participant 10] “Maybe instead of buttons, they could tap anywhere on the half of the screen. That would make it easier rather than to aim on the button. This will also help to mitigate the older folks who have difficulties to move on to the button” [Participant 2]	“Colours” for the asterisk [Participant 1] and for the buttons to be “round or bigger” [Participant 1]	“When press down on the star, right or correct, some positive words or pictures, smiling. Would be more interesting” [Participant 4] “If they hit the correct one, maybe there is a nice emoji. Wrong, maybe a crying emoji” [Participant 3]
Scoring functionality	Scoring was “very complicated” and that the scoring appealed only to “investigators or clinicians” [Participant 1]	No mention	“When they finish the game, can have instant results, instead of having to scroll down” [Participant 3]
Other possible functionalities	No mention	“What are the diseases you are going through if you have alcoholism? More information of what alcohol does to you in the short term and long term” [Participant 2]	No mention
Visual probe trial	No mention	“Just 3 seconds interval” [Participant 1] “You can request for 5-10 seconds interval delay” [Participant 1]	“Do it from slow, all the way to fast” [Participant 3]
Consideration of gaming elements	No mention	No mention	“Show the fastest speed and slowest speech in the result, make it like a game” [Participant 1] “Maybe you could have a board [1st, 2nd, 3rd)” [Participant 3]

### Phase 3

Gaming was the aspect where there was the greatest difference between health care professionals and patients. The professionals suggested the integration into the conventional attention bias modification app of performance-oriented rewards and storyboard gaming elements. With one exception, the inpatient participants were against the inclusion of gaming elements. The outpatient participants perceived that the inclusion of gaming elements was appropriate if the game was intended for individuals who are in abstinence. Table 4 provides a summary of the verbatim comments of the participants in accordance to the themes identified.

**Table 4:** Themes related to gamification elements for the existing app.

Themes	Health care professionals (n=10)	Inpatient participants (n=5)	Outpatient participants (n=5)
Performance-oriented gaming elements	“Lowest cost way of doing this app that will make users want to continue using it.” [Participant 2] “The feedback that you are doing better is kind of helpful for them” [Participant 9] “In between giving feedback about their performance; three times you press the correct thing, you are on a roll” [Participant 4] “As it motivates you to go into higher grades, either as an individual or as a group” [Participant 6] “Motivate them to continue playing the application” [Participant 11] “Bronze to silver to gold medals” [Participant 2] “Some achievement in the form of badges would help” [Participant 7] “Track their progress” “aware of the questions remaining” [Participant 8]	“Leader-board”, social gaming elements “Having connect with Facebook would allow you to see who else has participated” and “Levels” in the game [Participant 1]	“Results” of his or her performance [Participant 2] Time-pressure, levels and leaderboard were also chosen, as it allows for “competition” [Participant 2] “When you see the leaderboard, maybe I am here. I can do better to go up” [Participant 3]
Rewards (digital/real world)-based gaming elements	“Entice them to continue to play” [Participant 4]	No mention	“Can change for vouchers” [Participant 1] “Like voucher, exchange coffee. Get something realistic” [Participant 4]
Context (storyboard)	“Motivating” [Participant 7] “Map out the real-life experiences”, this giving “a reason in doing the exercises” [Participant 6] “More realistic and engaging” [Participant 9]	No mention	No mention
Against gamification	No mention	“Childish” [Participant 3] making the application to be more catered for “Kids” [Participant 1] “All these are game right? Part of games right? All these are addiction already” [Participant 2] “As a gamer, I could relate to Number 2, this is gaming addiction” [Participant 1]	“If I am using, I would not play game. When you are into drugs, you are in no mood to play games” [Participant 4] “When you are heavily using, you cannot be bothered by this. Especially when you are high” [Participant 5]

**Figure 2:**
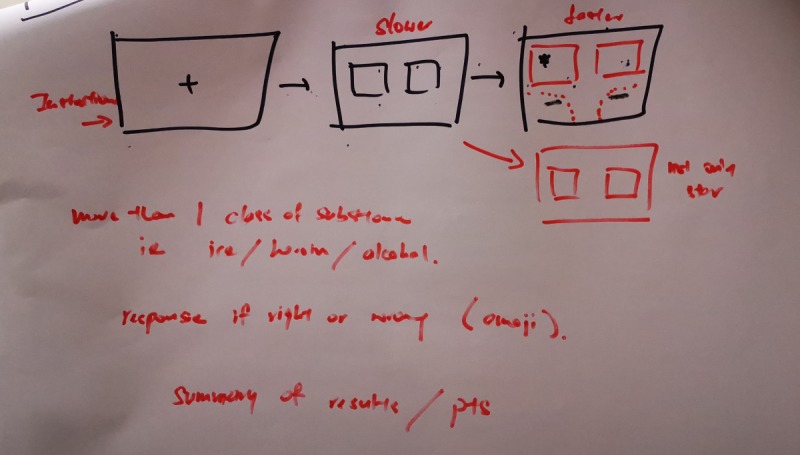
Patient-created proposal for design of the app.

Each of the six groups were able to discuss and sketch out a prototype of an app that would address the underlying limitations and include the solutions they have proposed. Figure 2 shows an example of the proposed design illustrated by one group of patients. Table 5 provides the selected verbatim comments of all groups of participants with regard to their sketched prototype. In each of the groups, one participant was asked to share about their prototype.

**Table 5:** Participants’ comments on their newly conceptualized prototypes (one participant was asked to share on behalf of the whole group).

Participants	Selected verbatim comments
Health care professionals (Group 1)	“We think that there should be a few levels of difficulties. It is like a training phase. The assessment time becomes shorter and shorter. The time becomes shorter and shorter. We think there should be a score. Encourage the person to hit a higher score. The rewards we were thinking about…Vouchers or privileges on the ward.”
Health care Professionals (Group 2)	“First thing would be instructions.” “Second part. There would be some motivation. You are doing good.” “After the training is done, task score. And the progress bar. How many percent completed.” “The pictures. We thought of different modalities. Not just the pictures. Sometimes virtual ones.” “Need a trial of how to do it.”
Inpatient participants (Group 1)	“Instead of using a cross, you can put a money sign.” “After you focus on a while, you could have different images.” “Secondly, instead of money, you could put a photo. A photo of your family. After you focus on the photo for a while, you drift off to an animated picture.” “Ask the user to type.”
Inpatient participants (Group 2)	“Family. Picture This is the family picture, wife and children picture. Drugs and alcohol. Which one you want to choose.” “Instead of someone else drinking, can be someone I know drinking.”
Outpatient participants (Group 1)	“The first square is the instruction manual. The second one is a prompt for ready. Then straight away go to the cross with 2 pictures. The last one is the start. And end with results. Maybe it starts off with a bit slower and then progress to fast.” “Maybe the first 20-30 pictures 0.8 seconds, then the next 0.6 seconds. Moderately decrease the speed instead of diving right in.”
Outpatient participants (Group 2)	“Introduction. The second column slightly slower than the tablet. 0.7-0.8 seconds. This one is 2 seconds. 1 seconds. Then you can random got different picture. Not only just come up star only. Can come up star with drug.”

In both groups of health care professionals, it was important that participants be allowed to have a training phase or practice before they undertake the actual task. Health care professionals also recommended the initial trial interval to be lengthened to 1000 milliseconds and gradually reduced to 500 milliseconds with time. Health care professionals also recommended the inclusion of scores and progress bars, to provide feedback and inform participants of their progress. Some health care professionals preferred the inclusion of animated images instead of static images. Inpatients wanted images that were animated or personalized for their substance of use. Participants also wanted a variety of images to appear on the screen, instead of being limited to merely two images. Like the design proposed by health care professionals, both groups of outpatient participants wanted to start with a lengthened trial interval, which was progressively shortened as the intervention progressed, for example, starting with a trial interval of 800 milliseconds for the first 20-30 set of images and then reducing it to 600 milliseconds. The rationale for this progressively decreasing trial interval duration was to allow participants of all age groups to become comfortable with the nature of the task. All participants emphasized the need to provide instructions prior to the commencement of the task. Both groups of participants also wanted the prototype to have bigger buttons to facilitate rapid response. All participants were able to complete the sketches of the prototype within the allocated time.

Participants were also shown images that were included in the existing app. Both health care professionals and participants shared their perspectives of the included images. Table 6 provides a summary of the verbatim comments of the participants in accordance with the drug categories.

**Table 6:** Suggestions of participants for images included in the app.

Image category	Health care professionals (n=10)	Inpatient participants (n=5)	Outpatient participants (n=5)
Alcohol	“Picture more relevant to the local context, for example, picture from coffee shop” [Participant 6] Not to focus on the “brands” of the alcohol [Participant 6]	“Hard liquor” was missing [Participants 1 and 2]	No need to include the “brand” of the alcohol [Participant 4] “More kind of hard liquor, such as whisky” [Participant 4]
Cannabis	“Some of the pictures were not familiar” [Participant 2]	A “barrel and aluminum foil” would be good [Participant 3] “Bottles” might not be so relevant, as they are synthetic cannabinoids [Participant 1]	“The bong is not the right bong” [Participant 2]
Heroin	“Straws” “pictures of the barrel and thin foils” [Participant 5] “Someone chasing the dragon, or someone using the main line” are the most relevant and triggering [Participant 3]	“The one with needle” [Participant 4]	“Syringe” image was perceived to be “very triggering” [Participant 1] “Nothing catches my eye except for this needle thing” [Participant 3] Pills “oxycodone” was not used locally (“Singapore people don’t abuse”). [Participant 1]
Stimulants	The images of the stimulant crystals might not be so relevant, as the crystals available locally are “of different quality” [Participant 2]	“Pure image of ICE^a^” [Participant 1]	“Don’t look like ICE at all” [Participant 4]

^a^ICE: methamphetamine (stimulant).

## Discussion

### Principal Findings

This is the first study to explore the principles of participatory design research for cognitive/attention bias modification interventions. When asked to identify the limitations in the existing app, common issues identified were those with the design, visual probe task, and included images. Outpatients were also concerned with the safety of administration of the intervention. In the brainstorming sessions, health care professionals made recommendations for enhancing the stimulus, mechanism of responding, and presentation of scores. Inpatient participants recommended the addition of functionalities such as information on the harms associated with the substance use and enhancements in the design, images, and tasks. Outpatient participants perceived the need to improve the images and presentation of the results and recommended the inclusion of gaming features. There were differences in opinions pertaining to the inclusion of gaming features, as only health care professionals endorsed their inclusion. In the last phase of the workshop, participants were tasked with the conceptualization of prototypes, and the commonality in the design was a gradual shortening of the interval for stimulus/image presentation.

Throughout all the co-design sessions, one of the main issues highlighted by participants was that the time interval for the presentation of the stimulus was too rapid. In the conventional app that we designed [23], we stipulated the following timings: 500 milliseconds for the presentation of the fixation cross, another 500 milliseconds for the presentation of the images, and 2000 milliseconds for the participant to respond before the trial goes on. A measure of 500 milliseconds was chosen as the time for the presentation of the stimulus, as most studies that have examined the reliability of the dot-probe task present cue stimuli 500 milliseconds prior to the appearance of the probe [26]. In the literature examining attentional bias modification for substance use disorder, there is a great variation in the timings of stimulus presentation. Charles et al [27] presented images for either 200 or 500 milliseconds to individuals with opioid use disorders, and they postulate that the short stimulus (200 milliseconds) helped in the evaluation of the automatic orientating, and the long stimulus (500 milliseconds) helped in the evaluation of controlled attentional processing. Other studies involving individuals with opioid disorders have used varied timings, ranging from a short stimulus of 200 milliseconds [28-32] to 500 milliseconds [29] and a long stimulus interval of 1500 milliseconds [15] to 2000 milliseconds [28,30-32]. In studies involving individuals with cannabis use disorder, stimulus intervals of 500 milliseconds [33] and 2000 milliseconds [34] have also been used. Studies involving participants with stimulant use disorders have, however, consistently used a stimulus interval of 500 milliseconds [35-37]. The aspects postulated by Charles et al [27] in their study on the evaluation of different attentional processes by short and long stimulus intervals have also been previously postulated by Robbins and Ehrman [38] and Field et al [39]. It is apparent that there is a wide variability in the stimulus interval timings in the published literature, despite all these studies having used the visual probe task. The findings from this study suggest that participants (both health care professionals and patients) prefer a slightly lengthened stimulus presentation interval (700-1000 milliseconds) and with the stimulus presentation interval gradually decreasing across the interventions. Taking into consideration the perspectives of our participants and the evidence to date in the published studies, future studies could vary the ratio of the stimuli that present as a long stimulus and a short stimulus (ie, at the start of the intervention, more trials are presented for a long stimulus duration, and across the days of interventions, this progressively decreases, so that there are more trials presented for a short stimulus duration). This helps ensure that both attentional processes (initial orientating and delayed disengagement) are being investigated.

One of the other limitations that were consistently highlighted throughout the workshops was a need for the stimulus images to be more relevant and personalized. The images included in the existing app were images extracted from the United States Drug Enforcement Agency Website together with some from the Singapore’s Central Narcotics Bureau’s website. Participants suggested that some of these images are not realistic enough for them and are not congruent with the images of the substances they have had used. To bridge this limitation, we could consider what Field et al [33] previously adopted. They had a separate group of 10 individuals, comprised of cannabis and noncannabis users, to rate the word stimulus on a “cannabis-relatedness” scale. They then used words that had the highest rating scores. No recent studies have replicated this study, and we propose that this should be considered in future research to ensure that the images included are relatable to the participants. Apart from relevance and relatedness, personalization of the images was suggested by inpatient participants. Although Field et al [40] reported that the poor reliability of the visual probe task could be attributed to the nature of the stimulus used and highlighted the need for personalization of images, a recent study by Jones et al [41] failed to demonstrate increased internal consistency of the visual probe task following personalization. Their study [41], which involved participants with alcohol use issues, presented participants with images related to their type of alcoholic beverage consumed rather than a broad range of alcohol images. Although an attractive option, it is evident from the published literature that personalization of images may not improve the reliability of the visual probe task and facilitate the detection of attentional biases. Moreover, there are privacy and practical constrains if personalization of the images is considered.

In this study, we found a discrepancy in the perspectives of health care professionals and patients with regard to the consideration of gaming elements. Health care professionals were open to the inclusion of gaming elements, whereas patients were cautious in considering gaming elements. It is extremely important to take into consideration the needs of patients, as they are the eventual final users. Allowing patients’ perspectives to take dominance over health care and academic perspective makes sense, given that it is increasingly recognized that patients bring unique insights and knowledge into the co-designing process [42]. This discrepancy in viewpoints could potentially be mitigated if health care professionals and patients are both included in the same co-design workshop. Unfortunately, this was not permitted by our ethical board, as there are concerns that patient participants might not be as vocal due to the presence of health care professionals. In our study, some patients acknowledged that gaming elements could enhance the existing task. The common gaming elements recommended by both health care professionals and patients were performance-oriented gaming elements (feedback, levels, etc) and rewards. In their review of gamified attention bias modification apps, Zhang et al [43] highlighted that apps previously evaluated included features like animations, sounds, feedback, and a point-scoring system for response time and difficulty. To some extent, our participants have endorsed similar gamification techniques, as the gaming elements that have been used previously could be easily clustered as performance-oriented gaming elements, according to a previous [24] taxonomy of gamification techniques. In the review by Zhang et al [43], only two of the four identified studies demonstrated that the gamified app was effective. Notably, the two studies that were efficacious were evaluating the same app. One study [44] that involved participants with alcohol use disorders did not find the game to be effective following the inclusion of gaming elements. Given our findings, it is pertinent for future studies to carefully consider the appropriate use of gaming elements, considering mainly the perspectives of patients, and to re-evaluate the app for its effectiveness. Future research should consider, in particular, the specific type of gaming strategy that would render the intervention more effective.

In our study, patients also highlighted the presence of safety concerns in the administration of such an attention bias modification app to individuals in the early stages of recovery. Their concerns are contrary to those of prior studies, which have introduced attention bias modification to individuals who were in the detoxification phase of their treatment [9,12]. Manning et al [12] highlighted that it is important to introduce bias modification early to capitalize on the stage of neural recovery. None of these prior studies have reported dropouts due to individuals relapsing into their substance of use. Nevertheless, the safety concerns highlighted by patients should be taken into consideration when executing a trial. It might be more appropriate to consider the administration of such an intervention among individuals in the later stages of their rehabilitation program or when they are out of their withdrawal phase. If the intervention must be administered to participants in the detoxification phase, only participants with low scores on their withdrawal scales should be selected to undertake such an intervention.

The main strength of this study is the principles of participatory design research for the coproduction of an attention bias modification app. The coproduced app not only included the perspectives of experts in the field of addiction (health care professionals), but also took into consideration the needs of the service users. Most of the existing attention bias and cognitive bias modification interventions are produced by either academic or commercial developers, and as highlighted by Zhang et al [15], there remains an apparent disconnect between academics, health care professionals, and software developers. This study has clearly bridged this gap in the research literature. In this study, we conducted the workshop in line with the principles of the Future Workshops.

Despite these inherent strengths, there are several limitations. It would have been most ideal to have both health care professionals and participants in the same workshop. However, we were unable to do so, as there is a possibility that the presence of health care professionals might make it uncomfortable for participants to share their inputs and perspectives. From the perspectives shared by the participants and from the results, it is evident that not all participants had the same understanding of the visual probe task paradigm, despite receiving the same introductory briefing. In addition, some of the themes identified such as cost were of importance, but the cost was not a theme that was commonly identified by both health care professionals and patients; therefore, it was not further explored. In our study, we showed participants examples of the various gamification strategies, as it is anticipated that our participants had a limited understanding of these techniques. There is a possibility that this might have resulted in some biases among individuals, but we have also taken steps to minimize this by ensuring that gamification techniques from all four categories, as previously highlighted by Hoffman et al [24], are shown. In addition, to minimize the risk of biases, in all three workshops, the facilitator explained each of the gamification strategies listed in the prior classification by Hoffman et al [24].

### Conclusions

To the best of our knowledge, this is the first study that has applied the principles of participatory design research for an attention bias modification intervention. Both health care professionals and patient participants provided insights on how the existing paradigm of the attention bias modification task could be enhanced to meet their needs. The results from this research will guide the development of an app that meets the specific needs of patients and is still based on a pre-existing validated task paradigm.

## Appendix

Multimedia Appendix 1 Demographic characteristics of patient participants.
