# Supplementary material for: Co-designing a Mobile Gamified Attention Bias Modification Intervention for Substance Use Disorders: Participatory Research Study
Source: JMIR Mhealth Uhealth. 2019 Oct 3;7(10):e15871. doi: 10.2196/15871 (PMC6797967; doi:10.2196/15871)
Supplement: Multimedia Appendix 1 [file mhealth_v7i9e15871_app1.pdf]

Appendix 1: Demographic Characteristics of Patient Participants

Demographic Characteristics	Outpatients	Inpatients
Nationality<br>1. Singaporean<br>2. Singapore PR / Others	 5 (100%)<br>0	 5 (100%)<br>0
Gender<br>1. Male<br>2. Female	 3<br>2	 5 (100%)<br>0
Marital Status<br>1. Single<br>2. Married<br>3. Divorced<br>4. Widowed / Others	 5 (100%)<br>0<br>0<br>0	 2<br>2<br>1<br>0
Race:<br>1. Chinese<br>2. Malay<br>3. Indian	 4<br>0<br>1	 2<br>0<br>3
Highest level of education<br>1. Primary Education and below<br>2. Secondary education<br>3. Junior College/Polytechnic/Technical studies/ Undergraduate and Postgraduate Studies	 0<br>4<br>1	 1<br>2<br>2
Employment Status<br>1. Unemployed<br>2. Part time<br>3. Full time	 4<br>0<br>1	 2<br>3<br>0
Housing Conditions<br>1. Homeless<br>2. Halfway house<br>3. 1 Room Rental Flat<br>4. 2 & 3 Room Flat<br>5. 4 & 5 Room Flat<br>6. Private Condo<br>7. Landed Property	 0<br>0<br>3<br>1<br>1<br>0<br>1	 1<br>0<br>0<br>1<br>3<br>0<br>0
*Substance Use:<br>1. Heroin<br>2. Other Opioids<br>3. Cannabis<br>4. Amphetamine<br>5. Cocaine<br>6. Alcohol	 2<br>1<br>2<br>4<br>0<br>2	 2<br>2<br>3<br>4<br>0<br>1
Days on treatment Facility (Average)	NA	4.7 days
Total abstinence period (Average)	14 months	NA

***Some participants have declared that they have used multiple drugs**
